# Downregulation of circular RNA hsa_circ_0000735 boosts prostate cancer sensitivity to docetaxel via sponging miR-7

**DOI:** 10.1186/s12935-020-01421-6

**Published:** 2020-07-22

**Authors:** Yisheng Gao, Jie Liu, Jing Huan, Fengyuan Che

**Affiliations:** 1 Guangzhou University of Chinese Medicine, Guangzhou, 510006 Guangdong China; 2 Department of Urology, Linyi People’s Hospital, Linyi, 276003 Shandong China; 3 Department of Acupuncture and Moxibustion, Linyi People’s Hospital, Linyi, 276003 Shandong China; 4 Department of Neurology, Linyi People’s Hospital, No. 27, East Section of Jiefang Road, Lanshan District, Linyi, 276003 Shandong China

**Keywords:** PCa, DTX, hsa_circ_0000735, miR-7

## Abstract

**Background:**

One of the main reasons for the failure of prostate cancer (PCa) treatment is the generation of chemoresistance. Circular RNA hsa_circ_0000735 (hsa_circ_0000735) is connected with the progression of cancer. Nevertheless, the role and regulatory mechanism of hsa_circ_0000735 in the resistance of PCa to docetaxel (DTX) are unclear.

**Methods:**

Expression levels of hsa_circ_0000735 and miR-7-5p (miR-7) in tissue samples and cells were examined via quantitative real-time polymerase chain reaction (qRT-PCR). The DTX sensitivity, viability, colony formation, cell cycle progression, and apoptosis of DTX-resistant PCa cells were determined via Cell Counting Kit-8 (CCK-8), cell colony formation, or flow cytometry assays. The levels of multidrug resistance protein 1 (MDR1) protein, cyclinD1, and B cell lymphoma 2 (bcl-2) were detected by western blotting. The interaction between hsa_circ_0000735 and miR-7 was verified via dual-luciferase reporter, RNA immunoprecipitation (RIP), and RNA pull-down assays. The role of hsa_circ_0000735 in vivo was validated through tumor formation experiments.

**Results:**

Hsa_circ_0000735 was upregulated and miR-7 was downregulated in DTX-resistant PCa tissues and cells. High hsa_circ_0000735 expression had a shorter overall survival. Both hsa_circ_0000735 knockdown and miR-7 mimic boosted DTX sensitivity, constrained viability, colony formation, cell cycle progression, and fostered apoptosis of DTX-resistant PCa cells. Also, hsa_circ_0000735 silencing elevated DTX sensitivity and repressed tumor growth in PCa in vivo. Mechanistically, hsa_circ_0000735 served as a sponge for miR-7. MiR-7 inhibition overturned hsa_circ_0000735 silencing-mediated impacts on DTX sensitivity and the malignant behaviors of DTX-resistant PCa cells.

**Conclusion:**

Hsa_circ_0000735 downregulation boosted PCa sensitivity to DTX and reduced tumor growth via sponging miR-7, providing a promising prognostic biomarker and therapeutic target for PCa.

## Highlight

1. Hsa_circ_0000735 was upregulated in DTX-resistant PCa tissues and cells.
2. Inhibition of hsa_circ_0000735 elevated DTX sensitivity and impeded the malignant behaviors of DTX-resistant PCa cells.
3. MiR-7 expression was reduced in DTX-resistant PCa tissues and cells.
4. Hsa_circ_0000735 acted as a sponge for miR-7 in DTX-resistant PCa cells.

## Background

Prostate cancer (PCa) is one of the leading causes of cancer-related deaths in men, ranking second in male malignancies [1]. About 1.6 million men are diagnosed with PCa each year, and about 366,000 men die from PCa [2]. Currently, PCa treatment mainly includes surgery, chemotherapy, androgen deprivation therapy, radiotherapy, and castration [3]. Although androgen deprivation therapy is effective in controlling metastatic PCa, most patients may develop castration-resistant prostate cancer (CRPCa) [4, 5]. Docetaxel (DTX)-based chemotherapy is the standard first-line therapy for CRPCa patients, which can prolong patient survival [6]. However, repeated DTX treatment can develop DTX resistance and reduce the treatment effect [7]. Therefore, understanding the latent mechanism of DTX resistance is critical for improving the prognosis of PCa patients [8].

Circular RNAs (circRNAs) are a type of non-coding RNAs with a covalently closed loop. They play vital roles in a variety of cellular physiological functions by acting as RNA-binding protein binding molecules, microRNA (miRNA) sponges, immune regulators, transcriptional regulators, or protein translation templates [9–11]. CircRNAs are also involved in tumor progression and chemoresistance in diverse cancers [12]. For instance, circRNA AKT3 impeded cisplatin chemosensitivity via regulating glycolysis balance through modulation of the miR-516b-5p/STAT3 axis in lung cancer cells [13]. Circular RNA hsa_circ_0000735 (hsa_circ_0000735) is derived from the P2RX1 gene (exons 2–8). Previous research revealed that hsa_circ_0000735 expression was connected with the clinical severity of NSCLC patients [14]. It has been reported that miRNAs can act as diagnostic, prognostic, and therapeutic biomarkers [15]. MiRNA-7-5p (miR-7) was proved to exert a repressive role in colorectal cancer [16], NSCLC [17], nasopharyngeal cancer [18], and so on. Furthermore, miR-7 could regulate tumor chemoresistance in some tumors. For example, miR-7 elevated temozolomide-resistant glioblastoma cell sensitivity and repressed stemness via targeting YY1 [19]. Also, miR-7 accelerated cervical cancer resistance to cisplatin by modulating the levels of B-cell lymphoma 2 (bcl-2) and PARP-1 [20]. However, it is indistinct whether hsa_circ_0000735 can regulate the resistance of PCa to DTX via interaction with miR-7.

In the present study, we demonstrated that hsa_circ_0000735 played a promotive role on the resistance of PCa to DTX. Furthermore, hsa_circ_0000735 knockdown elevated the sensitivity of PCa to DTX and reduced tumor growth through sponging miR-7.

## Materials and methods

### PCa specimens

The research was authorized and supervised by the ethics committee of Guangzhou University of Chinese Medicine. 50 paired PCa tissues and adjacent normal tissues were obtained from Guangzhou University of Chinese Medicine. Pathological examinations were performed according to the Solid Tumor Response Evaluation Criteria (RECIST), of which 23 specimens were sensitive and the rest were resistant. The clinicopathological features of PCa patients were displayed in Table 1. All participants did not receive hormone castration, immunotherapy, or radiotherapy before receiving DXT-based chemotherapy. Informed consents were obtained from all participants prior to analysis.

**Table 1:** The clinicopathological factures for PCa patients

Clinicopathological features	Number of cases
Age	
> 60 years	32
≤ 60 years	18
Tumor size (cm)	
> 2.5	29
≤ 2.5	21
Gleason score	
> 7	35
≤ 7	15
Clinical stage	
I + II	41
III	9
Preoperative PSA level (ng/mL)	
> 10	38
≤ 10	12
Lymph node metastasis	
Positive	19
Negative	31
DTX chemosensitivity	
Sensitive	23
Resistant	27

### Cell culture and treatment

PCa cell lines (PC-3 and DU145) and normal human prostatic epithelial cells RWPE-1 were bought from American Type Culture Collection (Manassas, VA, USA). All cells were cultured in Roswell Park Memorial Institute (RPMI)-1640 medium (Sigma, St Louis, MO, USA) supplemented with fetal bovine serum (10%, Gibco, Rockville, MD, USA) and penicillin/streptomycin (100 U/mL, Gibco) in an incubator with 5% CO_2_ at 37 °C. DTX-resistant PCa cell lines (PC-3/DTX and DU145/DTX) were obtained by gradually exposing the parental cell lines (PC-3 and DU145 cells) to a medium with increasing DTX doses (Sigma) for 6 months [21]. The starting concentration of DTX was 5 nM and the final concentration was 160 nM. The medium containing DTX was changed every 2–3 days. Moreover, 10 nM DTX was used for subsequent analysis.

### Cell transfection

Small interference RNA targeting hsa_circ_0000735 (si-circ-1 and si-circ-2) and matching control (si-NC), as well as lentivirus-mediated sh-hsa_circ_0000735 (sh-circ) and corresponding control (sh-NC), were achieved from Genepharma (Shanghai, China). MiR-7 mimic and inhibitor (miR-7 and anti-miR-7), as well as their negative controls (miR-NC and anti-NC), were purchased from Ribobio (Guangzhou, China). The oligonucleotides were transfected into DTX-resistant PCa cells through using Lipofectamine 3000 reagent (Life Technologies, Carlsbad, CA, USA).

### Quantitative real-time polymerase chain reaction (qRT-PCR)

The nuclear and cytoplasmic RNAs of DTX-resistant PCa cells were obtained with the PARIS kit (Life Technologies). Total RNA of tissue specimens and cells was obtained by using TRIzol reagent (Sigma). The complementary DNA for hsa_circ_0000735, P2RX1, miR-1182, miR-331-3p, miR-583, and miR-7 were synthesized using the High-Capacity complementary DNA Reverse Transcription Kit (Applied Biosystems, Foster City, CA, USA) or MicroRNA Reverse Transcription Kit (Applied Biosystems). The SYBR Green PCR Master Mix (Applied Biosystems) was mixed with primers for qRT-PCR analysis. The expression of hsa_circ_0000735, P2RX1, miR-1182, miR-331-3p, miR-583, and miR-7 was calculated using the 2^−ΔΔCt^ method [22]. The primers for miR-1182 (ID: MQPS0000444-1-100) were obtained from RiboBio, and the remaining primers were displayed in Table 2. Glyceraldehyde-3-phosphate dehydrogenase (GAPDH) or U6 small nuclear RNA (snRNA) snRNA was used as the internal control for hsa_circ_0000735, P2RX1, and miRNAs. 18S ribosomal RNA (rRNA) or U6 snRNA were used as the internal reference for cytoplasm and nucleus, respectively.

**Table 2:** Primers sequences used in this study

Gene	Forward primer sequence	Reverse primer sequence
hsa_circ_0000735	5′-GTGGAGTGGTTGGCATCACC-3′	5′-GAGAAACACCCACCTGAAGTTGA-3′
P2RX1	5′-ATGGTGCTGGTGCGTAATAAG-3′	5′-GGAAGACGTAGTCAGCCACA-3′
miR-331-3p	5′-GCGCCCCTGGGCCTATC-3′	5′-CGATGACCTATGAATTGACA-3′
miR-583	5′-CAAAGAGGAAGGTCCCATTAC-3′	5′-CAGTGCGTGTCGTGGAGT-3′
miR-7	5′-AAAACTGCTGCCAAAACCAC-3′	5′-GCTGCATTTTACAGCGACCAA-3′
GAPDH	F: 5′-GAAGGTGAAGGTCGGAGTC-3′	5′-GAAGATGGTGATGGGATTTC-3′
U6 snRNA	5′-GCTCGCTTCGGCAGCACA-3′	5′-GAGGTATTCGCACCAGAGGA-3′
18S rRNA	5′-GGAGTATGGTTGCAAAGCTGA-3′	5′-ATCTGTCAATCCTGTCCGTGT-3′

### Cell Counting Kit-8 (CCK-8) assay

For cytotoxic and half-maximal inhibitory concentration (IC50) value assessment, the transfected PC-3/DTX and DU145/DTX cells (2.0 × 10^3^) were cultured in cell medium with different concentrations of DTX (5, 10, 20, 40, 80, or 160 nM) for 48 h. For cell viability analysis, the transfected PC-3/DTX and DU145/DTX cells (2.0 × 10^3^) were seeded into 96-well plates with cell medium (10 nM DTX) for 24 h, 48 h, or 72 h. Thereafter, the CCK-8 solution (10 μL, Beyotime, Shanghai, China) was added to each well and incubated for 2 h. The absorbance at 450 nm was measured with a microplate reader (Bio-Rad, Hercules, CA, USA).

### Cell colony formation assay

The transfected PC-3/DTX and DU145/DTX cells (1.0 × 10^2^ cells/well) were seeded into 6-well plates and then treated with 10 nM DTX when the cell was attached to the wall. The medium was replaced every 2 days. After culture for 10 days, the cells were fixed with paraformaldehyde (4%, Beyotime) and then stained with and crystal violet (0.1%, KeyGen, Jiangsu, China). The number of colonies (> 50 cells) was counted and photographed through a light microscope (Olympus, Tokyo, Japan).

### Flow cytometry assay

For cell cycle progression analysis, the transfected PC-3/DTX and DU145/DTX cells were seeded in dishes (6 cm) and disposed with 10 nM DTX. After about 80% confluence, the cells were collected and fixed with ethanol (75%). Next, the cells were stained with propidium iodide (PI) (500 μL, Sigma) in the presence of RNase A (20 μg/mL, Sigma). Thereafter, the cells were washed with phosphate buffer solution (PBS) and the distribution of the cells was determined with the FACScan flow cytometry (Gallios, Beckman, USA).

The Annexin V-fluorescein isothiocyanate (FITC)/PI apoptosis detection kit (KeyGen) was utilized for cell apoptosis assessment. In brief, the transfected PC-3/DTX and DU145/DTX cells were cultured for 48 h after 10 nM DTX treatment. Then, the cells (5 × 10^5^) were resuspended in binding buffer (1×) and then stained with Annexin V-FITC (10 μL) and PI (5 μL). The samples were analyzed through the FACScan flow cytometry (Gallios).

### Western blotting

The RIPA buffer (Beyotime) containing protease inhibitor (1%, Sigma) was used to extract total protein from tissue specimens and cells. Western blotting was performed according to a previous study [23]. The primary antibodies were exhibited as blows: anti-multidrug resistance protein 1 (MDR1) (ab170904, 1:2000), anti-cyclinD1 (ab40754, 1:2000), anti-bcl-2 (ab182858, 1:2000), and anti-GAPDH (ab9484, 1:1000). Goat anti-rabbit IgG (ab97051, 1:1000) was used as a secondary antibody. The immunoblot was visualized via using the enhanced chemiluminescence solution (Beyotime) and GAPDH was used as a loading control. All antibodies were bought from Abcam (Cambridge, MA, USA).

### RNA immunoprecipitation (RIP) assay

The RIP RNA-Binding Protein Immunoprecipitation Kit (Millipore, Billerica, MA, USA) was used for RIP analysis. In short, PC-3/DTX and DU145/DTX cells transfected with si-circ-1 or si-NC were lysed with RIP buffer. Then, the lysates were incubated with protein A/G magnetic beads conjugated to Ago2 or IgG antibodies. The immunoprecipitated RNAs were obtained using the RNeasy Mini Kit (Qiagen, Valencia, CA, USA). The enrichment of hsa_circ_0000735 was evaluated via qRT-PCR.

### RNA pull-down assay

In short, the hsa_circ_0000735 probe or Oligo probe (Sigma) was incubated with C-1 magnetic beads (Life Technologies) to generate the probe-coated beads. Thereafter, the probe-coated beads were incubated with sonicated PC-3/DTX and DU145/DTX cells. The RNA complexes were extracted with the RNeasy Mini Kit (Qiagen). The levels of miR-1182, miR-331-3p, miR-583, and miR-7 were evaluated with qRT-PCR.

### Dual-luciferase reporter assay

The binding sites between hsa_circ_0000735 and miR-7 were predicted by circBank and circinteractome databases. The sequences of wild type (WT) hsa_circ_0000735 and mutant (MUT) hsa_circ_0000735 (MUT1, MUT2, and MUT1 + MUT2) were inserted into the pmirGLO luciferase vectors (GeneCreat, Wuhan, China). The luciferase reporter vectors were cotransfected into PC-3/DTX and DU145/DTX cells with miR-NC or miR-7, respectively. The luciferase activities were determined with the luciferase reporter assay kit (Promega, Madison, WI, USA).

### Tumor formation experiments

The protocols of tumor formation experiments were approved by the ethics committee of Guangzhou University of Chinese Medicine. In brief, DU145/DTX cells (2.0 × 10^6^ cells/0.2 mL PBS) infected with lentivirus-mediated sh-circ or matching control (sh-NC) were subcutaneously injected into the right flank of 15 BALB/c nude mice (5-week-old, Experimental Animal Center, Shanghai, China), which 5 mice in the sh-circ group and 10 mice in the sh-NC group. Nude mice were intraperitoneally injected with PBS or DTX (10 mg/kg) twice a week when the tumor volume reached 100 mm^3^. All nude were fed under Specific Pathogen Free conditions. The volume of tumors was measured once a week using a caliper. After 28 days, all mice were killed for subsequent analysis. Tumors volume was calculated with the equation: Volume =  (length × width^2^)/2.

### Statistical analysis

The data of the experiments in vitro were expressed as mean ± standard deviation and derived from 3 replicate experiments. The normal distribution was determined by a Kolmogorov–Smirnov test. GraphPad Prism 6.0 software was utilized for statistical analysis. Differences were deemed significant if *P* < 0.05. The independent Student’s *t* test was used for the evaluation of the differences between two independent groups. The homogeneity of the variance was analyzed with an F-test. One-way variance analysis with hoc post Turkey test was utilized to compare the differences among more groups. The survival curves were generated using the Kaplan–Meier method with log-rank test.

## Results

### Hsa_circ_0000735 was upregulated in DTX-resistant PCa tissues and cells, and high hsa_circ_0000735 expression had a poor prognosis

**Fig. 1:**
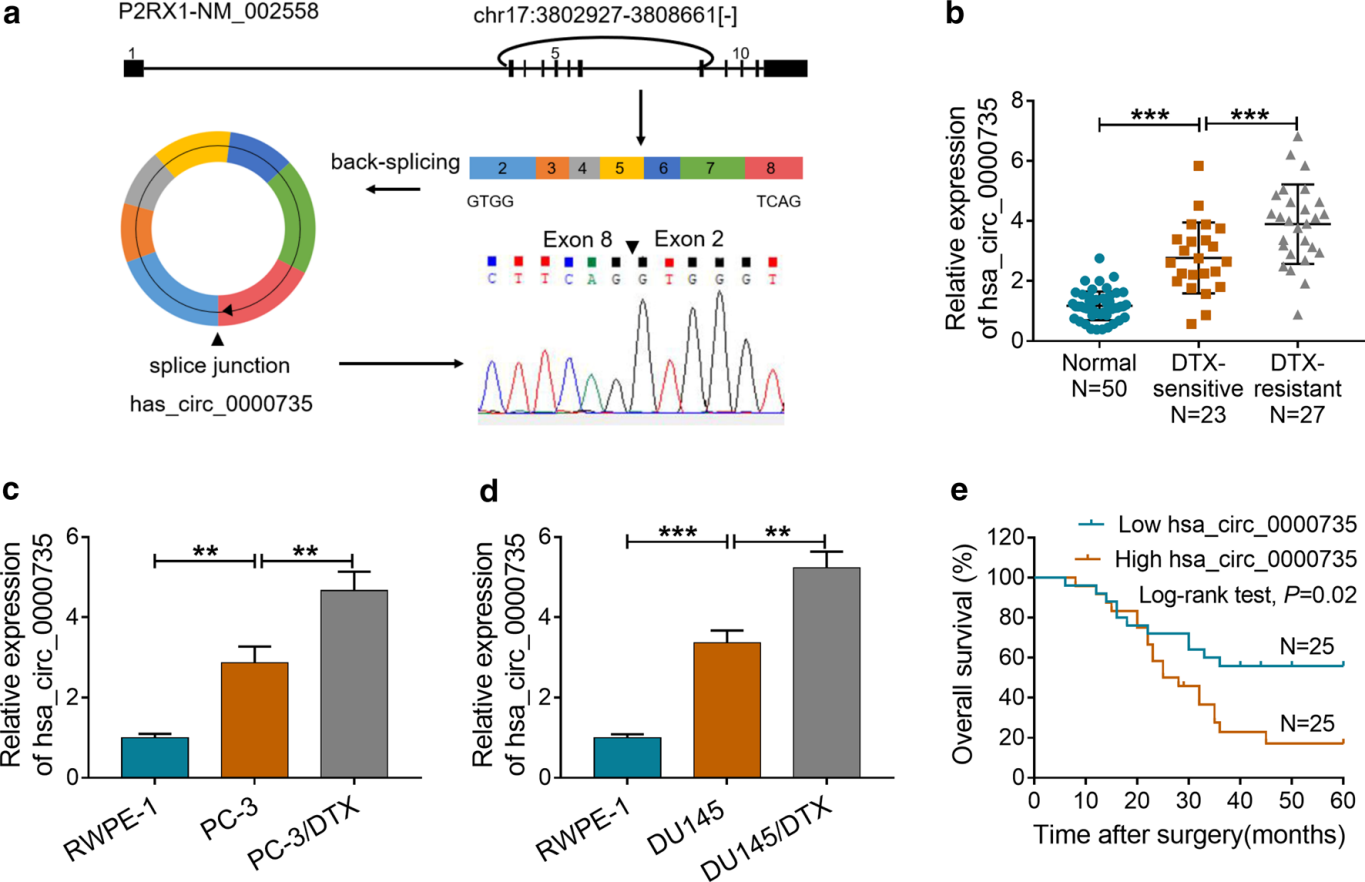
Expression pattern of hsa_circ_0000735 in DTX-resistant PCa tissues and cells. **a** The schematic exhibited the circularizing of P2RX1 exons 2-8 into the form of hsa_circ_0000735 by head-to-tail. The splice junction of hsa_circ_0000735 was verified through RT-PCR and Sanger sequencing. **b** The expression levels of hsa_circ_0000735 in 23 DTX-sensitive PCa tissues, 27 DTX-resistant PCa tissues, and 50 adjacent normal tissues were examined through qRT-PCR. **c**, **d** The expression levels of hsa_circ_0000735 in PC-3, DU145, PC-3/DTX, DU145/DTX, and RWPE-1 cells were evaluated by qRT-PCR. **e** Kaplan–Meier survival curves of overall survival for patients with high or low hsa_circ_0000735 expression after DTX treatment. The median value of hsa_circ_0000735 expression was deemed as the cutoff value. **P < 0.01 and ***P < 0.001

Hsa_circ_0000735 is derived from the P2RX1 gene (exons 2–8), whose spliced mature sequence length is 738 bp. Results of Sanger sequencing validated the head-to-tail splicing in the RT-PCR product of hsa_circ_0000735 (Fig. 1a). To explore the role of hsa_circ_0000735 in the resistance of PCa to DTX, we first examined the expression of hsa_circ_0000735 in 23 DTX-sensitive PCa tissues, 27 DTX-resistant PCa tissues, and 50 adjacent normal tissues. The results presented that hsa_circ_0000735 expression was overtly boosted in DTX-sensitive PCa tissues in comparison to the adjacent normal tissues. And hsa_circ_0000735 expression was higher in DTX-resistant PCa tissues than that in DTX-sensitive PCa tissues (*P* < 0.001, Fig. 1b). Subsequently, we constructed DTX-resistant PCa cells (PC-3/DTX and DU145/DTX) and assessed the expression of hsa_circ_0000735 in PC-3/DTX and DU145/DTX cells. As displayed in Fig. 1c, d, the expression of hsa_circ_0000735 was apparently elevated in PCa cells (PC-3 and DU145) relative to the RWPE-1 cells. Moreover, hsa_circ_0000735 expression was visibly higher in PC-3/DTX and DU145/DTX cells compared to their parental cells (*P* < 0.01 and *P* < 0.001, Fig. 1c, d). After DTX treatment, PCa patients with high hsa_circ_0000735 level had lower overall survival compared with PCa patients with low hsa_circ_0000735 level (*P* = 0.02, Fig. 1e). Collectively, these results suggested that high hsa_circ_0000735 expression in PCa might be involved in the resistance of PCa to DTX.

### Hsa_circ_0000735 silencing elevated DTX-resistant PCa cell sensitivity to DTX

**Fig. 2:**
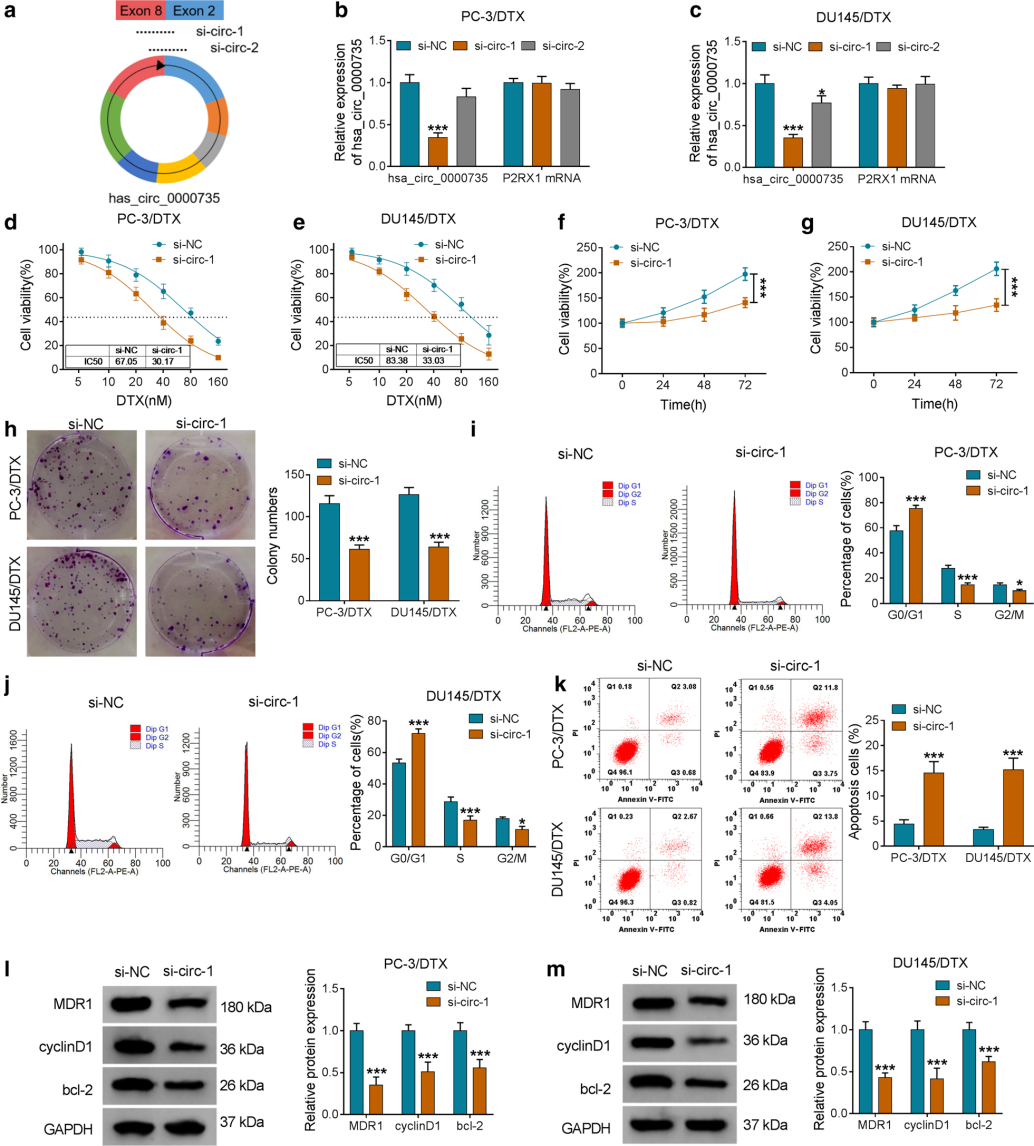
Influence of hsa_circ_0000735 knockdown on the sensitivity of DTX-resistant PCa cells to DTX. **a** The illustration exhibited siRNAs (si-circ-1 and si-circ-2) that target the junction of hsa_circ_0000735. **b**, **c** QRT-PCR results for P2RX1 mRNA and hsa_circ_0000735 in PC-3/DTX and DU145/DTX cells transfected with si-NC, si-circ-1, or si-circ-2. **d**, **e** The viability of PC-3/DTX and DU145/DTX cells transfected with si-NC or si-circ-1 in the presence of DTX at the different concentrations (5, 10, 20, 40, 80, or 160 nM) for 48 h was determined through CCK-8 assay. **f**–**k** The viability, colony formation, cell cycle progression, and apoptosis of PC-3/DTX and DU145/DTX cells transfected with si-NC or si-circ-1 under DTX (10 nM) treatment were assessed with CCK-8, cell colony formation, or flow cytometry assays. **l**, **m** The levels of MDR1, cyclinD1, and bcl-2 in PC-3/DTX and DU145/DTX cells transfected with si-NC or si-circ-1 under DTX (10 nM) treatment were assessed via western blotting. *P < 0.05 and ***P < 0.001

In consideration of the upregulation of hsa_circ_0000735 in DTX-resistant PCa tissues and cells, we further explored the function of hsa_circ_0000735 in the resistance of PCa to DTX. We designed two siRNAs (si-circ-1 and si-circ-2) targeting the unique splice junction of hsa_circ_0000735 (Fig. 2a). The results exhibited that hsa_circ_0000735 expression was clearly reduced in PC-3/DTX and DU145/DTX cells after si-circ-1 transfection compared to the control si-NC, but there was no effect on the expression of liner P2RX1 mRNA (*P* < 0.05 and *P* < 0.001, Fig. 2b, c). CCK-8 assay presented that hsa_circ_0000735 knockdown boosted the sensitivity of PC-3/DTX and DU145/DTX cells to DTX and reduced the IC50 value of PC-3/DTX and DU145/DTX cells in contrast to the si-NC group (Fig. 2d, e). Subsequently, we analyzed the impacts of hsa_circ_0000735 inhibition on the malignant behaviors of DTX-resistant PCa cells treated with DTX (10 nM). CCK-8 assay indicated that hsa_circ_0000735 silencing repressed cell viability in PC-3/DTX and DU145/DTX cells (*P* < 0.001, Fig. 2f, g). Cell colony formation assay manifested that reduced hsa_circ_0000735 expression constrained the colony formation ability of PC-3/DTX and DU145/DTX cells (*P* < 0.001, Fig. 2h). Flow cytometry assay indicated that silenced hsa_circ_0000735 expression enhanced the number of PC-3/DTX and DU145/DTX cells in G0/G1 stage and reduced the number of PC-3/DTX and DU145/DTX cells in S and G2/M stages, indicating that hsa_circ_0000735 silencing arrested cell cycle progression (*P* < 0.05 and *P* < 0.001, Fig. 2i, j). Also, the apoptotic rate of PC-3/DTX and DU145/DTX cells was boosted after si-circ-1 transfection (*P* < 0.001, Fig. 2k). Additionally, the levels of MDR1, cyclinD1, and bcl-2 were reduced in PC-3/DTX and DU145/DTX cells after si-circ-1 transfection (*P* < 0.001, Fig. 2l, m). Taken together, these findings manifested that hsa_circ_0000735 knockdown could boost cell sensitivity to DTX in DTX-resistant PCa cells.

### Hsa_circ_0000735 acted as a sponge for miR-7 in DTX-resistant PCa cells

**Fig. 3:**
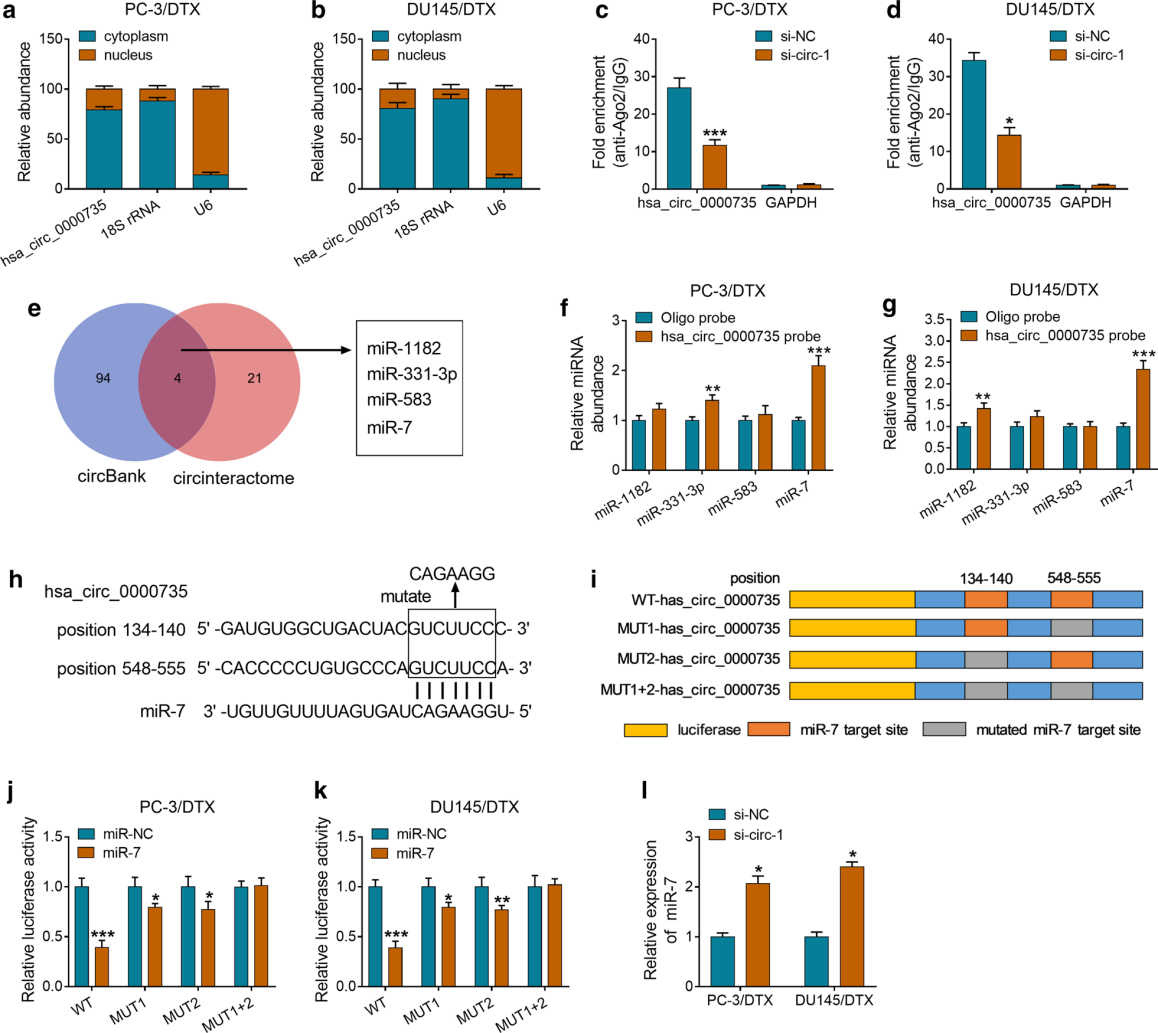
Hsa_circ_0000735 served as a sponge for miR-7 in DTX-resistant PCa cells. **a**, **b** QRT-PCR was conducted to detect the levels of hsa_circ_0000735 in cytoplasm and nucleus of PC-3/DTX and DU145/DTX cells. **c**, **d** RIP assay was carried for the assessment of hsa_circ_0000735 level in PC-3/DTX and DU145/DTX cells transfected with si-circ-1 or si-NC. **e** Schematic illustration the overlap of the target miRNAs of hsa_circ_0000735 predicted by circBank and circinteractome databases. **f**, **g** RNA pull-down assay was carried out by using hsa_circ_0000735 probe to pull down miR-1182, miR-331-3p, miR-583, and miR-7. **h** The binding sites between hsa_circ_0000735 and miR-7 were exhibited. **i** Schematic diagram of the luciferase reporters, which contained the WT-hsa_circ_0000735, MUT1-hsa_circ_0000735, MUT2-hsa_circ_0000735, or MUT1 + 2-hsa_circ_0000735 sequences. **j**, **k** Dual-luciferase reporter assay was conducted to analyze the luciferase intensity of the luciferase reporters in PC-3/DTX and DU145/DTX cells transfected with miR-NC or miR-7. **l** The expression of miR-7 in PC-3/DTX and DU145/DTX cells transfected with si-circ-1 or si-NC was analyzed via qRT-PCR. *P < 0.05, **P < 0.01, and ***P < 0.001

Based on the above findings, we further exported the regulatory mechanism of hsa_circ_0000735 in the resistance of PCa to DTX. Firstly, we assessed the levels of hsa_circ_0000735 in cytoplasm and nucleus of PC-3/DTX and DU145/DTX cells. The results exhibited that hsa_circ_0000735 was enriched in cytoplasm, indicating that hsa_circ_0000735 could play a role via binding to a miRNA (Fig. 3a, b). RIP assay revealed that hsa_circ_0000735 was pulled down by anti-Ago2 in PC-3/DTX and DU145/DTX cells and the enrichment of hsa_circ_0000735 was lower after si-circ-1 transfection (*P* < 0.05 and *P* < 0.001, Fig. 3c, d). Then, we predicted the latent miRNAs that could bind to hsa_circ_0000735 through circBank and circinteractome databases. Both circBank and circinteractome databases displayed that miR-1182, miR-331-3p, miR-583, and miR-7 had base pairs complementary to hsa_circ_0000735 (Fig. 3e). Next, the RNA pull-down assay was conducted to verify whether these miRNAs could directly bind hsa_circ_0000735. We discovered that miR-7 was overtly pulled down by hsa_circ_0000735 probe in PC-3/DTX and DU145/DTX cells compared to the control group (*P* < 0.01 and *P* < 0.001, Fig. 3f, g). The binding sites between miR-7 and hsa_circ_0000735 were exhibited in Fig. 3h. In addition, the reporter vectors containing WT-hsa_circ_0000735, MUT1-hsa_circ_0000735, MUT2-hsa_circ_0000735, or MUT1 + 2-hsa_circ_0000735 were cotransfected into PC-3/DTX and DU145/DTX cells together with miR-7 or miR-NC (Fig. 3i). Dual luciferase reporter assay revealed that miR-7 overexpression distinctly suppressed the luciferase activity of the reporter vectors containing WT-hsa_circ_0000735, MUT1-hsa_circ_0000735, and MUT2-hsa_circ_0000735 compared to the miR-NC group, but the luciferase activity of the reporter vectors containing WT-hsa_circ_0000735 was overtly lower. However, there was no distinct difference in the reporter vectors containing MUT1 + 2-hsa_circ_000073 (*P* < 0.05, *P* < 0.01, and *P* < 0.001, Fig. 3j, k). Also, the downregulation of hsa_circ_0000735 contributed to the expression of miR-7 in PC-3/DTX and DU145/DTX cells (*P* < 0.05, Fig. 3l). Taken, these findings manifested that hsa_circ_0000735 served as a sponge for miR-7 in DTX-resistant PCa cells.

### MiR-7 elevated the sensitivity of DTX-resistant PCa cells to DTX

**Fig. 4:**
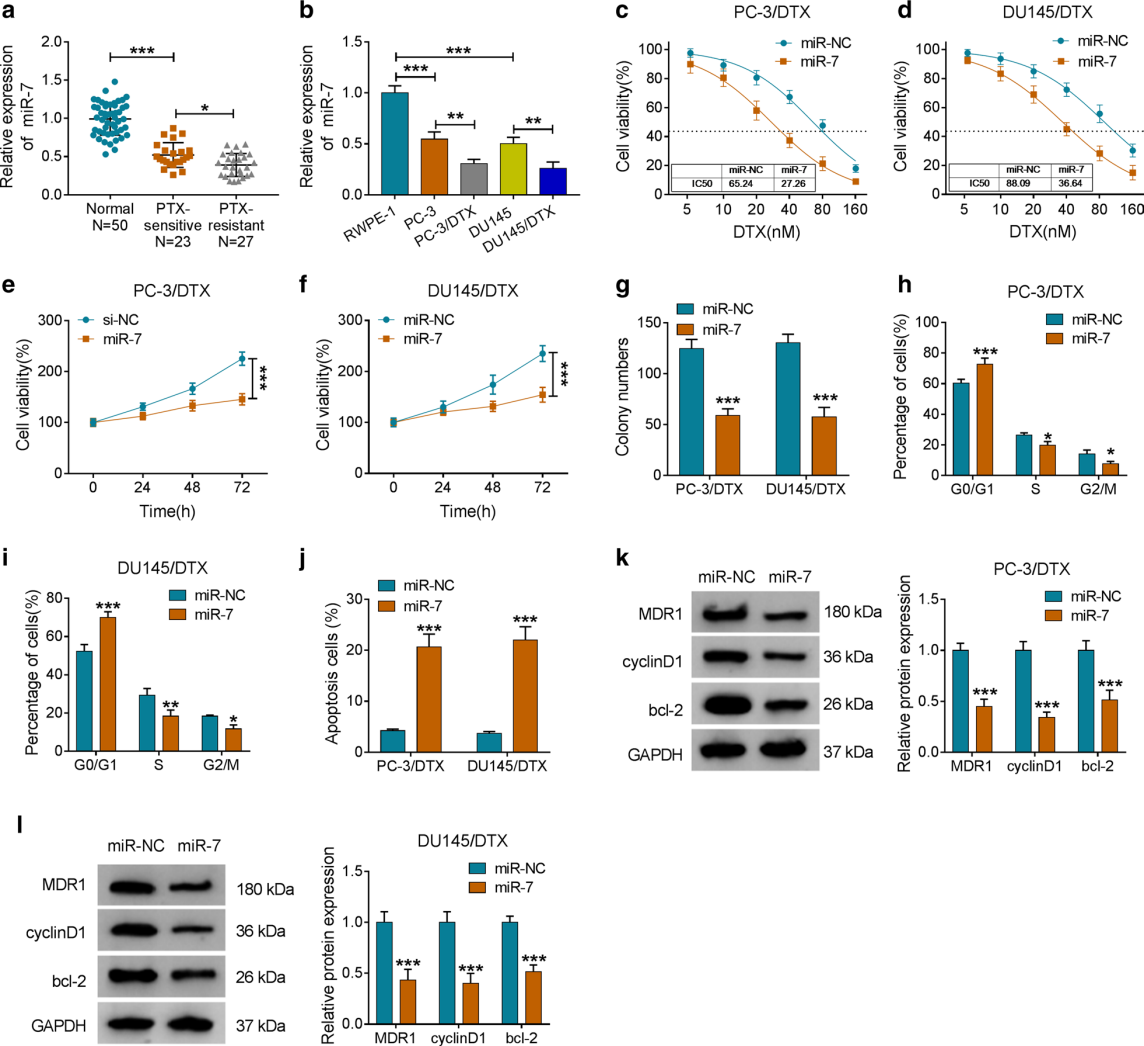
Effects of miR-7 mimic on the sensitivity of DTX-resistant PCa cells to DTX. **a** QRT-PCR was conducted to examine the expression of miR-7 in 23 DTX-sensitive PCa tissues, 27 DTX-resistant PCa tissues, and 50 adjacent normal tissues. **b** QRT-PCR was performed to analyze the expression of miR-7 in PC-3, DU145, PC-3/DTX, DU145/DTX, and RWPE-1 cells. **c**, **d** CCK-8 assay was carried out to evaluate the sensitivity and IC50 value of PC-3/DTX and DU145/DTX after miR-7 or miR-NC transfection. **e**–**j** The viability, cell cycle progression, and apoptosis of PC-3/DTX and DU145/DTX cells transfected with miR-7 or miR-NC under DTX (10 nM) treatment were assessed with CCK-8, cell colony formation, or flow cytometry assays. **k**, **l** Western blotting was performed to examine the levels of MDR1, cyclinD1, and bcl-2 in PC-3/DTX and DU145/DTX cells transfected with miR-7 or miR-NC under DTX (10 nM) treatment. *P < 0.05, **P < 0.01, and ***P < 0.001

To assess the function of miR-7 in the resistance of PCa to DTX, we detected the expression of miR-7 in 23 DTX-sensitive PCa tissues, 27 DTX-resistant PCa tissues, and 50 adjacent normal tissues. Compared to the adjacent normal tissues, the expression of miR-7 was markedly decreased in DTX-sensitive PCa tissues, and miR-7 expression was lower in DTX-resistant PCa tissues than that in the DTX-sensitive PCa tissues (*P* < 0.05 and *P* < 0.001, Fig. 4a). Consistently, miR-7 was downregulated in PC-3 and DU145 cells compared with the RWPE-1 cells, and its expression was apparently lower in PC-3/DTX and DU145/DTX cells when compared with their parental cells (*P* < 0.05, *P* < 0.01, and *P* < 0.001, Fig. 4b). Furthermore, miR-7 overexpression enhanced PC-3/DTX and DU145/DTX cell sensitivity to DTX and reduced the IC50 value of the cells (Fig. 4c, d). Enhanced miR-7 expression impeded the viability and colony formation ability of PC-3/DTX and DU145/DTX cells after DTX (10 nM) treatment (*P* < 0.001, Fig. 4e–g). Also, miR-7 mimic induced cell cycle arrest and apoptosis of PC-3/DTX and DU145/DTX cells under DTX (10 nM) treatment (*P* < 0.05, *P* < 0.01, and *P* < 0.001, Fig. 4h–j). And miR-7 elevation markedly decreased the levels of MDR1, cyclinD1, and bcl-2 in PC-3/DTX and DU145/DTX cells treated with DTX (10 nM) (*P* < 0.001, Fig. 4k, l). These data indicated that miR-7 overexpression increased cell sensitivity to DTX in DTX-resistant PCa cells.

### Inhibition of miR-7 overturned hsa_circ_0000735 knockdown-mediated influence on DTX sensitivity of DTX-resistant PCa cells

**Fig. 5:**
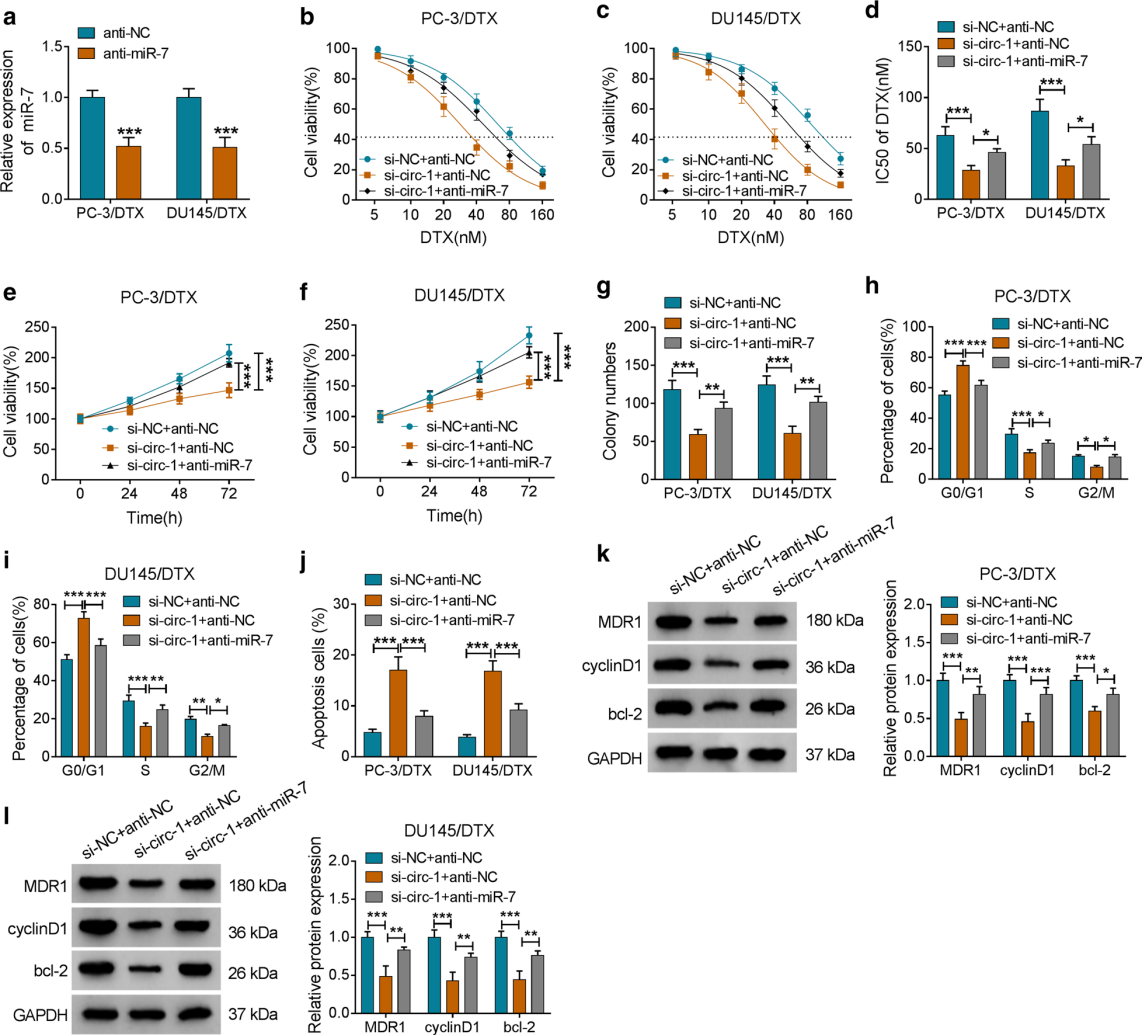
Hsa_circ_0000735 regulated the resistance of PCa to DTX through miR-7. **a** The expression of miR-7 in PC-3/DTX and DU145/DTX cells transfected with anti-miR-NC or anti-NC was evaluated by qRT-PCR. **b**–**l** PC-3/DTX and DU145/DTX cells were transfected with si-NC + anti-NC, si-circ-1 + anti-NC, or si-circ-1 + anti-miR-7, respectively. **b**–**d** The DTX sensitivity and IC50 value of PC-3/DTX and DU145/DTX cells were assessed with CCK-8 assay. **e**–**j** The viability, colony formation, cell cycle progression, and apoptosis of PC-3/DTX and DU145/DTX cells after DTX (10 nM) treatment were determined via CCK-8, cell colony formation, or flow cytometry assays. **k**, **l** The levels of MDR1, cyclinD1, and bcl-2 of PC-3/DTX and DU145/DTX cells treated with DTX (10 nM) were detected by western blotting. *P < 0.05, **P < 0.01, and ***P < 0.001

Given that hsa_circ_0000735 acted as a sponge for miR-7 in DTX-resistant PCa cells, we further investigated whether hsa_circ_0000735 exerted its role in DTX-resistant PCa cells via miR-7. We observed that miR-7 was downregulated in PC-3/DTX and DU145/DTX cells after anti-miR-7 transfection compared to the control group (*P* < 0.001, Fig. 5a). Moreover, both the elevation of DTX sensitivity and the decrease of IC50 value of PC-3/DTX and DU145/DTX cells mediated by hsa_circ_0000735 silencing were partly reversed by miR-7 inhibition (*P* < 0.05 and *P* < 0.001, Fig. 5b–d). Furthermore, the inhibitory effect of hsa_circ_0000735 downregulation on the viability and colony formation of PC-3/DTX and DU145/DTX cells were recovered by miR-7 inhibitor (*P* < 0.01 and *P* < 0.001, Fig. 5e–g). Also, the downregulation of miR-7 abolished hsa_circ_0000735 inhibition-mediated impacts on cell cycle progression and apoptosis of PC-3/DTX and DU145/DTX cells (*P* < 0.05, *P* < 0.01, and *P* < 0.001, Fig. 5h–j). In addition, decreased miR-7 expression abrogated the suppressive influence of hsa_circ_0000735 silencing on the levels of MDR1, cyclinD1, and bcl-2 of PC-3/DTX and DU145/DTX cells (*P* < 0.05, *P* < 0.01, and *P* < 0.001, Fig. 5k, l). Therefore, these results indicated that hsa_circ_0000735 exerted its role via miR-7 in DTX-resistant PCa cells.

### Hsa_circ_0000735 inhibition boosted tumor sensitivity to DTX in vivo

**Fig. 6:**
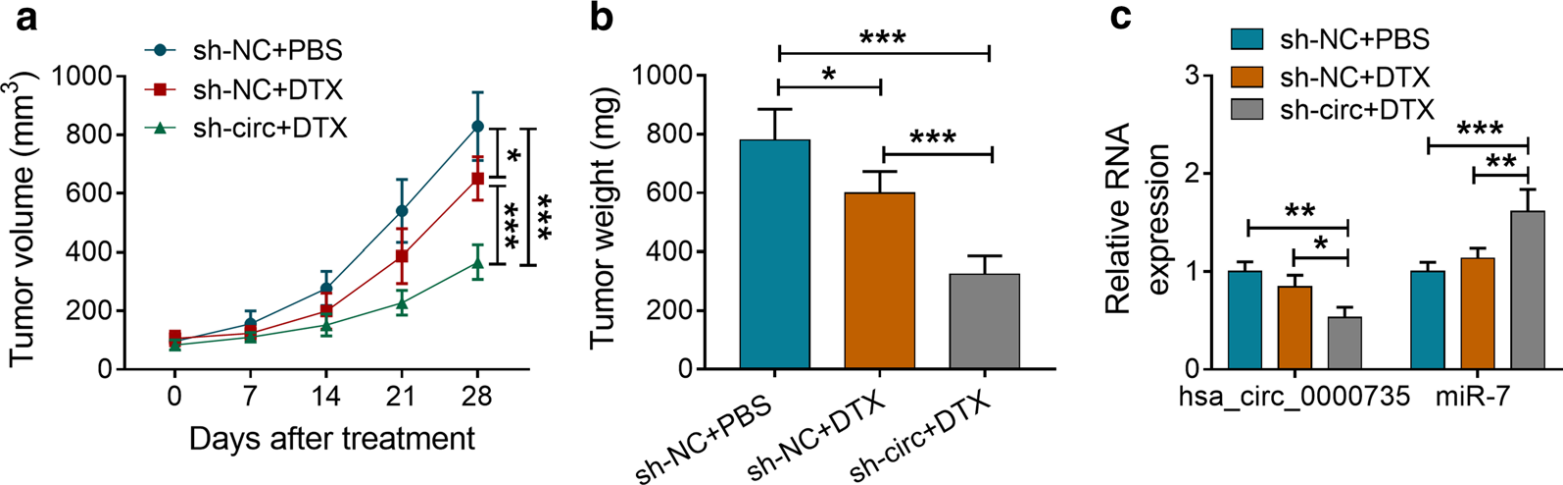
Hsa_circ_0000735 silencing elevated tumor sensitivity to DTX in vivo. **a** Tumor volume of mice in sh-NC + PBS, sh-NC + DTX, and sh-circ + DTX groups was measured once a week through a caliper. **b** Tumor weight of mice in sh-NC + PBS, sh-NC + DTX, and sh-circ + DTX groups was assessed after injection 28 days. **c** The expression of hsa_circ_0000735 and miR-7 in tumor tissues of mice of the sh-NC + PBS, sh-NC + DTX, or sh-circ + DTX groups was determined with qRT-PCR. *P < 0.05, **P < 0.01, and ***P < 0.001

To further validate the role of hsa_circ_0000735 on the resistance of PCa cells to DTX in vivo, we subcutaneously injected DU145/DTX cells with or without stable hsa_circ_0000735 knockdown into the nude mice and then treated with PBS or DTX. The data manifested that tumor growth (volume and weight) was reduced in the sh-NC group after DTX treatment compared to the sh-NC group without DTX treatment, but hsa_circ_0000735 knockdown increased tumor sensitivity to DTX and further inhibited tumor growth (*P* < 0.05 and *P* < 0.001, Fig. 6a, b). Furthermore, hsa_circ_0000735 expression was overtly decreased in tumor tissues of mice in the sh-circ group after DTX treatment compared to the sh-NC group with or without DTX treatment, while miR-7 expression was distinctly elevated (*P* < 0.05, *P* < 0.01, and *P* < 0.001, Fig. 6c). These results indicated that hsa_circ_0000735 inhibition elevated tumor sensitivity to DTX in vivo.

## Discussion

With the development of high-throughput technologies, more and more circRNAs have been found to exert important regulatory roles in different types of treatment resistance in human tumors [12]. Moreover, circular RNAs are promising diagnostic and prognostic markers, as well as therapeutic targets [24–27]. Increased research indicated that circRNAs could regulate the chemoresistance of PCa. Greene et al. revealed that decreased has_circ_0004870 expression might exert a vital role in enzalutamide resistance development in CRPCa [28]. Another study claimed that has_circ_0001427 boosted the enzalutamide sensitivity and repressed invasion in CRPCa cells via regulation of the miR-181c-5p/Arv7 pathway [29]. Xiang et al. reported that circRNA UCK2 repressed PCa progression via enhancing enzalutamide chemosensitivity through modulating the miR-767-5p/TET1 axis [30]. Herein, we discovered that hsa_circ_0000735 was upregulated in DTX-resistant PCa tissues and cells, and high hsa_circ_0000735 expression possessed a shorter overall survival, indicating that hsa_circ_0000735 was a promising prognostic biomarker for PCa. Also, hsa_circ_0000735 silencing elevated DTX-resistant PCa cell sensitivity to DTX in vitro and in vivo. Report of Li et al. indicated that hsa_circ_0000735 elevation contributed to the malignant behaviors of NSCLC cells [14]. These data indicated that hsa_circ_0000735 played an accelerative in the resistance of PCa to DTX and could act as a prognostic biomarker for PCa. MDR1, also known as P-glycoprotein, plays an important role in multidrug resistance [31]. CyclinD1 is an important promoter of the cell cycle [32]. Bcl-2 contributes to the survival of cancer cells by inhibiting pro-apoptotic protein activation [33]. In our study, hsa_circ_0000735 silencing repressed viability, colony formation, induced cell cycle arrest and apoptosis of DTX-resistant PCa cells under DTX treatment in vitro. Moreover, hsa_circ_0000735 knockdown repressed the levels of MDR1, cyclinD1, and bcl-2 in DTX-resistant PCa cells under DTX treatment. These data further proved that hsa_circ_0000735 could elevate DTX-resistant PCa cell sensitivity to DTX.

It has been proved that circRNAs regulated the chemoresistance of tumors via sponging miRNAs [12, 29, 30]. In this study, we discovered that hsa_circ_0000735 severed as a sponge for miR-7 by dual-luciferase reporter, RIP, and RNA pull-down assays. Lai et al. unmasked that miR-7 was downregulated in doxorubicin-resistant small cell lung cancer cells, and miR-7 enhancement repressed doxorubicin-induced homologous recombination repair through inhibiting Rad51 and BRCA1 expression, which elevated the doxorubicin sensitivity of doxorubicin-resistant small cell lung cancer cells [34]. Moreover, miR-7 could increase cell sensitivity to temozolomide and impede cell stemness in temozolomide-resistant glioblastoma cells through downregulating YY1 [19]. Also, miR-7 expression could be repressed by lncRNA KCNQ1OT1 in oxaliplatin-resistant hepatocellular cancer cells, which accelerated cell resistance to oxaliplatin [35]. Paccez et al. revealed that dihydroartemisinin could constrain PCa progression via repressing AXL expression through inducing miR-7 and miR-34a expression [36]. Moreover, miR-7 overexpression inhibited KLF4 expression through KLF4-miR-7 feedback looping to impede PCa cell growth [37]. Herein, miR-7 expression was reduced in DTX-resistant PCa tissues and cells, and miR-7 mimic enhanced the DTX sensitivity of DTX-resistant PCa cells. Our data indicated that miR-7 reduced the resistance of PCa to DTX, which was consistent the previous studies [36, 37]. Furthermore, miR-7 inhibitor abolished hsa_circ_0000735 inhibiton-mediated influence on DTX sensitivity of DTX-resistant PCa cells. Therefore, we concluded that hsa_circ_0000735 modulated the sensitivity of PCa to DTX through sponging miR-7.

## Conclusion

In summary, we proved that hsa_circ_0000735 silencing could enhance PCa sensitivity to DTX via sponging miR-7, providing an evidence for hsa_circ_0000735 as a prognostic biomarker and therapeutic target for PCa. In the future, the downstream pathway of the hsa_circ_0000735/miR-7 axis can be studied.

## Data Availability

All data generated or analysed during this study are included in this published article.
